# Polarization and orientation of retinal ganglion cells *in vivo*

**DOI:** 10.1186/1749-8104-1-2

**Published:** 2006-10-13

**Authors:** Flavio R Zolessi, Lucia Poggi, Christopher J Wilkinson, Chi-Bin Chien, William A Harris

**Affiliations:** 1 Department of Physiology, Development and Neuroscience, University of Cambridge, Cambridge, UK; 2 Sección Biología Celular, Departamento de Biología Celular y Molecular, Facultad de Ciencias, Universidad de la República, Montevideo, Uruguay; 3 Department of Neurobiology and Anatomy, University of Utah School of Medicine, Salt Lake City, UT, USA

## Abstract

In the absence of external cues, neurons *in vitro *polarize by using intrinsic mechanisms. For example, cultured hippocampal neurons extend arbitrarily oriented neurites and then one of these, usually the one nearest the centrosome, begins to grow more quickly than the others. This neurite becomes the axon as it accumulates molecular components of the apical junctional complex. All the other neurites become dendrites. It is unclear, however, whether neurons *in vivo*, which differentiate within a polarized epithelium, break symmetry by using similar intrinsic mechanisms. To investigate this, we use four-dimensional microscopy of developing retinal ganglion cells (RGCs) in live zebrafish embryos. We find that the situation is indeed very different *in vivo*, where axons emerge directly from uniformly polarized cells in the absence of other neurites. *In vivo*, moreover, components of the apical complex do not localize to the emerging axon, nor does the centrosome predict the site of axon emergence. Mosaic analysis in four dimensions, using mutants in which neuroepithelial polarity is disrupted, indicates that extrinsic factors such as access to the basal lamina are critical for normal axon emergence from RGCs *in vivo*.

## Introduction

A key step in neuronal morphogenesis is the emergence of correctly oriented axons and dendrites. The cellular and molecular mechanisms that determine how one neurite is selected to become the axon while the others become dendrites have been studied extensively in conditions where this problem is most approachable experimentally, namely *in vitro *[1]. If hippocampal cells are cultured soon after their final mitotic divisions, multiple neurites emerge simultaneously at seemingly random orientations. From these young multipolar neurons, one neurite then begins to elongate preferentially, marking the beginning of polarization. It becomes the axon [2], and as it grows it inhibits the other neurites from becoming axons. They become dendrites instead. The inhibitory signal relies on the activities of the small GTPases Rac/Cdc42 and Rho [3,4] and on the localized inactivation of GSK-3β [5,6]. Proteins normally associated with the apical junctional complexes of epithelial cells, such as Par-3, Par-6 and atypical protein kinase C (aPKC) have a role in polarization *in vitro*. Aided by adenomatous polyposis coli and KIF3A (a kinesin superfamily protein), proteins that travel along microtubules, these apical components accumulate at the tips of growing axons. Interference with the activity of any of these proteins compromises polarization [7-9]. The centrosome, acting as a microtubule organizing center, also has a role in axon formation *in vitro *[10], and recent evidence suggests that its position determines of the site of axon emergence [11].

In dissociated cell cultures, neurons develop in the presence of very scarce external cues, and so must perforce break symmetry intrinsically. *In vivo*, however, neurons are generated within a highly oriented three-dimensional neuroepithelium. In such a situation, differentiating neurons may depend on external cues for polarization. In support of this idea, Rolls and Doe [12] demonstrated that, in *Drosophila *mutants lacking the apical junction components Par-3, Par-6 or aPKC, neurons in the central nervous system *in vivo *are nevertheless appropriately oriented. This study raises two important questions. First, are the dissimilarities in these results due to differences between vertebrates and invertebrates or are they due to differences between the situation *in vitro *and that *in vivo*? Second, if extrinsic cues polarize neurons *in vivo*, how is this done in such a way that neurons become appropriately oriented?

Retinal ganglion cells (RGCs) are an excellent model system with which to study the above questions. Restricted to a layer adjacent to the inner basement membrane, RGCs show 'typical' neuronal polarity, with basally oriented axons and apically oriented dendritic trees. More than a century ago, Ramón y Cajal made observations of the embryonic chick retina and drew differentiating 'RGCs' with bipolar morphologies, including a retracting apical process and an axon extending from the basal side [13]. In line with this, Hinds and Hinds [14], in their serial electron microscopic studies of the developing mouse retina, suggested that the axons of RGCs arise from the basal process of neuroepithelial-like precursors. By injecting Lucifer yellow into differentiating RGCs in *Xenopus *embryos, Holt [15] showed that RGC axons almost always emerge from the basal pole of the cell. Halfter and Schurer [16] found that disruption of the inner basement membrane of the developing chick retina led to aberrant RGC axon outgrowth. Together, these studies suggest a relationship between axon orientation in differentiating RGCs and the basal surface of the neuroepithelium. However, in all of these studies the images were static and the analysis depended on the cells' having already assumed the beginnings of RGC morphology, including the formation of a primordial axon. To understand what is going on when neurons first polarize, it is essential to be able to follow single cells from their final mitosis to the time when they extend a definitive axon. Only through such studies is it possible to learn, for example, whether differentiating RGCs *in vivo *go through an early multipolar phase in which they put out several exploratory neurites before they stabilize one as the axon.

In the zebrafish, through the use of transgenes that drive fluorescent proteins under the control of an enhancer-promoter from the *ath5 *gene (*atoh7*) and four-dimensional (4D) microscopy, it is possible to view the differentiation of RGCs *in vivo *from their final mitosis at the apical surface, through to the initiation of their axonal and dendritic processes [17]. We took advantage of these innovations to show that RGCs send out axons directly from their basal surface in the absence of other neurites emerging from the cell. Tracing the movements of different apical markers, such as junctional complex or centrosomal proteins, in these transgenic retinas revealed that these components remain in the retracting apical process while the axon extends from the opposite (basal) pole of the differentiating cell body of the RGC.

To look for extrinsic cues in polarizing RGCs *in vivo*, we used two mutants in which the polarity of the retinal neuroepithelium is disrupted: *nagie oko *(*nok*) and *heart and soul *(*has*) [18]. Detailed 4D analyses of wild-type RGCs in mutant environments show that the polarization and orientation of RGCs is determined by the local orientation of the neuroepithelium, including factors such as the presence of a basal lamina. RGCs without access to either the inner or outer basal lamina during their differentiation go through a multipolar phase that precedes polarization, as they do *in vitro*. Our results provide strong evidence for an extrinsic influence in RGC polarization.

## Materials and methods

### Animals

Zebrafish were maintained and bred at 26.5°C, and embryos were raised at 28.5°C. The mutant lines used were: *nagie oko *(*nok*^*m*227^, a kind gift from Dr Jarema Malicki) and *heart and soul *(*has*^*m*567^, a kind gift from Dr Salim Abdelilah-Seyfried). Both represent null alleles of the respective genes. Two transgenic lines were generated in our laboratory: *Tg(pBatoh7:gap43-gfp)*^*cb*1 ^('*ath5:gap-gfp*') and *Tg(pBatoh7:gap43-rfp)*^*cb*2 ^('*ath5:gap-rfp*'). They express a fluorescent protein (enhanced green fluorescent protein (EGFP) or monomeric red fluorescent protein 1 (mRFP1), respectively) fused to the GAP43 N-terminal palmitoylation signal, under the control of the zebrafish *ath5 *promoter (comprising 7 kilobases of genomic sequence upstream of the *ath5 *start codon). For some experiments we used a transgenic line expressing a cytoplasmic form of EGFP under the control of the *ath5 *promoter ('*ath5:gfp*', a kind gift from Dr Ichiro Masai) [19]. The *ath5:gap-gfp *transgenic line was crossed with carriers of both mutations used, to generate an F_1 _generation from which mutant embryos expressing GAP-EGFP in RGCs could be obtained, namely *nok*^*m*227 ^× *Tg(pBatoh7:gap43-gfp)*^*cb*1 ^and *has*^*m*567 ^× *Tg(pBatoh7:gap43-gfp)*^*cb*1^.

### Constructs, expression of exogenous proteins and morpholino treatment of embryos

Constructs used to inject into living embryos were as follows: *ath5:gap-gfp*, *ath5:gap-rfp*, GFP-zcentrin and Par3-GFP [20]. For generating the *ath5:gap-gfp *and *ath5:gap-rfp *expression vectors, a fragment containing 7 kilobases from the 5' regulatory region of the zebrafish *ath5 *gene [19] has been subcloned upstream to either the GAP-EGFP or GAP-mRFP coding regions [21]. The promoter and coding region were subcloned in the IsceI pBSII SK+ vector, kindly provided by Dr Jochen Wittbrodt [22]. For the GFP-zcentrin construct, pCJW263 is a pCS2+-based plasmid in which zebrafish centrin is joined in-frame, 3' to EGFP. It was created in the following steps. The *Sal*I site of pCS2P+ was removed by digestion then infilling with Klenow enzyme and religation. This plasmid was cut with *Bam*HI, the 5' overhangs filled in with Klenow enzyme and then cut again with *Xba*I. This fragment was ligated with the *Afe*I-*Xba*I fragment of pEGFP-C2 (Clontech, Mountain View, CA, USA) that contains the EGFP coding region and multiple cloning sites to create the vector pCS2P+EGFPN. Zebrafish centrin was amplified by PCR using IMAGE clone 5899515 as template and these primers: 5'-TTGGATCCTCATGGCGTCCGGCTTCAGGAAAAGC-3' (forward) and 5'-TTCTCGAGGTACAGATTGGTTTTCTTCATAATCCG-3' (reverse). The PCR product was digested with *Bgl*II and *Xho*I and ligated with *Bgl*II- and *Sal*I-cut pCS2P+EGFPN. GFP-zcentrin mRNA was transcribed from the Sp6 promoter of pCJW263, linearized with *Not*I, using the mMessage machine *in vitro *transcription kit (Ambion, Austin, TX, USA). RNA was purified with the RNeasy RNA purification kit (Qiagen GmbH, Hilden, Germany).

For transient expression of fluorescent proteins, embryos were injected with either plasmid containing the gene of interest, under the control of a general (cytomegalovirus) promoter or the RGC progenitor-specific (*ath5*) promoter, or with mRNA transcribed *in vitro*. DNA injections were made into the cell at the one-cell stage, whereas mRNA injections were done into the yolk at the one-cell to four-cell stage, using a micromanipulator-mounted micropipette and a Picospritzer microinjector. The maximum volumes for injection were 2.5 nl into the cell and 5 nl into the yolk. For the *ath5 *promoter-driven constructs, the plasmids were injected together with meganuclease *I-Sce-I *at a concentration of 10 ng/μl into one-cell-stage embryos, as described [22]. For stable transgenesis, embryos expressing the fluorescent protein at the correct location were selected and raised to sexual maturity; transgenic carriers were identified by outcrossing to wild-type fish.

Morpholinos diluted in water were injected into the yolk at the one-cell to eight-cell stage. Morpholinos used were as follows: anti-*nok*, translation blocking (MORPH1116, Open Biosystems); anti-*slit1b*, translation blocking (LDHMO2, 5'-GCTCGGTGTCCGGCATCTCCAAAAG-3', designed by L Hutson and C-BC) and anti-*slit1a*, splice blocking (S1ASDMO1, 5'-GAAATAAACTCACAGCCTCTCGGTG-3', designed by M Hardy and C-BC). The ideal amount to be injected was determined by analyzing a range of concentrations. We found, for the Slit1b morpholino, different responses in different genetic backgrounds, but these were corrected for by adjusting the amount injected, resulting in the same reproducible phenotypes. For the analysis of the data we took into account only embryos that had received the same relative amount of morpholino (namely, 2 to 3 ng for the *ath5:gap-gfp *line and 6 to 8 ng for the *ath5:gap-rfp *line) that did not cause obvious effects in cell survival.

### Live imaging of whole-mounted embryos

Embryo processing and 4D imaging were performed as described previously [17]. Usually, stacks about 100 μm thick, composed of sections separated by 1 μm, were taken every 5 to 10 minutes during an average period of 20 to 24 hours. To avoid damaging the embryos, we maintained the power of the lasers at a minimum (typically 12 to 20%). The 4D data thus obtained were processed and analyzed with Volocity (Improvision, Coventry, UK). Unless stated otherwise in the figure legends, the images shown are maximum-intensity projections of all or most of the confocal stack. Quantifications described in the text were made using Volocity, Openlab (Improvision, Coventry, UK) or ImageJ (National Institutes of Health) software.

### *In situ *immunostaining, neuronal retrograde labeling and transmission electron microscopy

Cryosections were made at 10 μm thickness from 4% paraformaldehyde-fixed, OCT-mounted zebrafish embryos. Blocking was for 30 minutes at 20 to 23°C, in 10% heat-inactivated goat serum (HIGS), 1% bovine serum albumin, 0.2% Triton X-100 in PBS. Primary and secondary antibodies were incubated for 1 hour at room temperature, diluted as described below. For whole-mount immunostaining, embryos (grown in 0.003% phenylthiourea) were fixed overnight in 4% paraformaldehyde in PBS, and all subsequent washes were performed in PBS containing 0.2% Triton X-100. Further permeabilization was achieved by incubating the embryos in 0.25% trypsin-EDTA in Hanks balanced salt solution for 15 to 25 minutes at 0°C. Blocking and antibody dilution was as for sections. Antibodies were incubated for at least 36 hours at 4°C, with occasional shaking.

The primary antibodies, diluted in the blocking solution, were as follows: Zn-5, 1/100 to 1/500 dilution (mAb anti-Ben/DM-GRASP, specific for RGCs in the differentiating neural retina; Zebrafish International Resource Center (ZIRC), Eugene, OR, USA; Zpr-2, 1/100 dilution (mAb specific for retinal pigment epithelium (RPE); ZIRC); anti-laminin 1, 1/60 dilution (poly-clonal antibody (pAb), L9393; Sigma, St Louis, MO, USA); anti-Tau 1, 1/500 dilution (pAb; Dr Itzhak Fischer); anti-aPKC-ζ, 1/250 to 1/500 dilution (pAb; New England Biolabs, Hitchin, UK); and anti-α-catenin, 1/2,000 dilution (pAb, Sigma). Secondary antibodies used were goat anti-mouse IgG and goat anti-rabbit IgG Cy3-conjugated (Chemicon, Temecula, CA, USA), goat anti-mouse IgG and goat anti-rabbit IgG Alexa 488-conjugated (1/1,000 to 1/2,000 dilution; Molecular Probes, Eugene, OR, USA). When necessary, phalloidin-Texas Red (Molecular Probes) was mixed with the secondary antibody. Nuclei were counterstained with 4',6-diamidino-2-phenylindole. For retrograde labeling of RGCs, we micro-injected small amounts of 1,1'-dioctadecyl-3,3,3',3'-tetra-methylindocarbocyanine perchlorate (DiI; Molecular Probes) diluted in chloroform into the right tectum of fixed zebrafish embryos 79 hours after fertilization (hpf). After a variable period of incubation (room temperature or 4°C), embryos were counterstained and processed for confocal imaging.

Photomicrography was performed with either a laser confocal system as described or with Nikon fluorescence microscopes, equipped with cooled charge-coupled device (CCD) Hamamatsu Orca cameras and automated *z*-drive and fluorescence shutters. Acquisition of *z*-stacks and deconvolution were performed with Openlab software.

For transmission electron microscopy, embryos were dissected rapidly and fixed for 4 hours at 4°C in 4% glutaraldehyde/0.3% H_2_O_2_, in an isotonic phosphate buffer.

After being processed for transmission electron microscopy with the use of standard procedures, ultrathin sections were imaged in an FEI-Philips CM100 system.

### Retinal cell culture and blastomere transplantation

Eyes extracted from zebrafish embryos just before or around the onset of RGC differentiation (25 to 28 hpf) were dissociated with trypsin-EDTA at 28.5°C, and cells were seeded at a density of eight eyes per dish in 13 mm coverslip-bottom dishes covered with laminin. After incubation for 1 hour in 200 μl of L15 medium containing 10% FCS, 3 to 4 ml of L15 supplemented with N-2 (Invitrogen, Paisley, Renfrewshire, UK) was added. Cells were then either kept at 28.5°C until needed or used immediately for time-lapse analysis. Cultures from *has *mutants (and their wild-type controls) were made from 30 to 32 hpf embryos, as cell differentiation seems to be delayed in the mutants. Time-lapse studies of cultured retinal cells were conducted in a Nikon TE300 inverted fluorescent microscope, equipped with a Hamamatsu Orca AG cooled CCD digital camera and automated *z*-drive and shutters. For data acquisition and analysis we used the Openlab software, taking stacks of images (1 μm steps) every 10 to 20 minutes.

For immunostaining, cells were fixed by adding to the culture medium an equal amount of 4% paraformaldehyde, 15% sucrose in 1 × PBS, for 1 hour at room temperature. After being washed, cells were permeabilized with 0.1% Triton X-100 in PBS, and immunostaining and photomicrography were performed as described for cryosections.

With the aim of generating genetic mosaic embryos, we transplanted 10 to 40 blastomeres from labeled embryos (expressing *ath5:gap-gfp *transgene and/or injected with dextran-rhodamine or H2B-YFP mRNA (YFP being yellow fluorescent protein) to obtain a general labeling) into the animal poles of unlabeled blastulas. In brief, embryos were embedded in 2% methylcellulose on a coverslip, and usually cells were transferred from one donor to up to six hosts with a glass micropipette as described [23]. Embryos were incubated as usual, keeping the donor apart when necessary to identify the mutants morphologically.

## Results

### Zebrafish retinal ganglion cells polarize intrinsically *in vitro*

**Figure 1:**
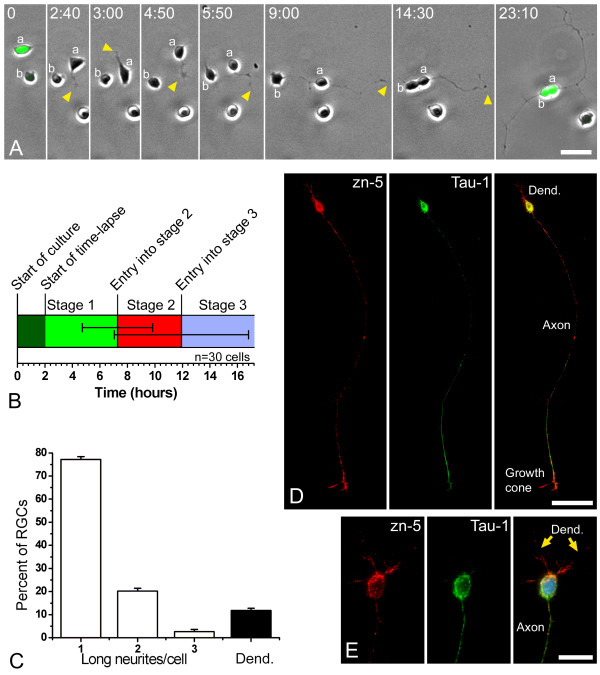
Retinal ganglion cells (RGCs) polarize *in vitro *after a period of plastic behavior. **(a) **Time-lapse analysis of dissociated *ath5:gfp*-expressing retinal cells in culture (Additional file 1). At the start of the time-lapse, cell 'a' expressed a higher level of GFP and was already in late stage 1 of differentiation (namely forming long filopodia), whereas cell 'b' showed a lower fluorescence and was at early stage 1. A short time later cell 'a' started to generate short processes ('neurites'; arrowheads), indicating the onset of stage 2. One of the neurites formed a growth cone at time point 4 minutes 30 seconds and started to grow faster at the beginning of stage 3. After the cell bodies made contact, near the end of the sequence, cell 'b' also seemed to extend an axon-like neurite. Time is shown in hours:minutes. Scale bar, 15 μm. **(b) **Graphic representation of the timing of *in vitro *differentiation of zebrafish RGCs, where 30 cells were followed by time-lapse video microscopy. The horizontal lines in the middle of the bar represent the standard deviation from the transitions between stages 1 and 2 and between stages 2 and 3. **(c) **Analysis of the morphology of the RGCs (Zn5-positive cells) after 24 hours in culture. *n *= 100 cells, in three independent cell-culture experiments. 'Long neurites' are longer than three cell diameters. **(d, e) **Cultured RGCs at stage 4 (24 hours *in vitro*), labeled with the RGC-specific antibody Zn-5 (red), an anti-Tau-1 antibody (green) and 4',6-diamidino-2-phenylindole in (e). Scale bars, 30 μm (d) and 10 μm (e).

To establish whether zebrafish RGCs, like mammalian hippocampal neurons, have the ability to polarize intrinsically, we explanted dissociated cells from 26 to 28 hpf transgenic zebrafish retinas that express EGFP in RGCs under the control of the RGC precursor-specific *ath5 *promoter. Time-lapse analyses of these cells revealed initial stages of polarization that, although much more rapid, are fundamentally similar to those described for rat hippocampal cells [2] (Figure 1a,b). Immediately after explantation, *ath5:gfp*-positive cells are round and show intense surface activity in the form of pseudopodia and short filopodia. In some cases, a rapid circular movement of pseudopodia, known as 'circus movements' [24], was seen. This 'stage 1' lasts about 7 hours (Figure 1b). 'Stage 2' is shorter (lasting about 4 hours) and is characterized by the appearance of several short neurites that alternately elongate and retract, so that cells in stage 2 often have two or more neurites at the same time. Suddenly, and marking the start of 'stage 3', one neurite shows a conspicuous growth cone and begins to grow faster (Figure 1a and Additional file 1). By 24 hours, most of the RGCs appear unipolar (Figure 1c). 'Stage 4' is usually visible from the second day of culture (Figure 1c) in RGCs with one long Tau-1-positive axon-like neurite and a few short, Tau-1-negative neurites branching at the opposite pole of the cell body (Figure 1d,e). Our next question was whether we could observe a similar process *in vivo*.

### Polarization of retinal ganglion cells *in vivo *is distinct from that *in vitro*

**Figure 2:**
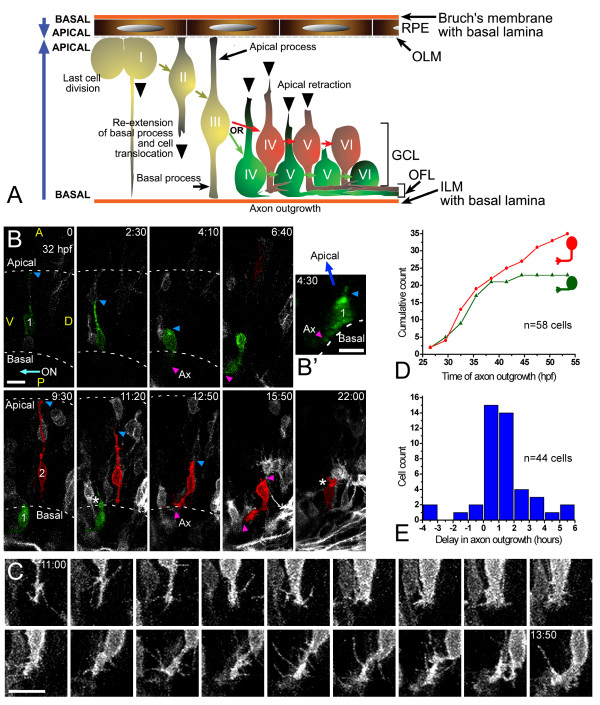
Retinal ganglion cell (RGC) polarization *in vivo *seems restricted by the environment. **(a) **Summary of the observed behaviors of normal RGCs *in vivo*. GCL, ganglion cell layer; ILM, inner limiting membrane; OFL, optic fiber layer; OLM, outer limiting membrane. **(b) **Time-lapse confocal (four-dimensional (4D)) analysis of an *ath5:gap-gfp*-injected embryo, showing two differentiating cells that undergo apical retraction and axonogenesis (Additional file 2). Cell 1 (green) shows no basal process when forming the axon (see view rotated 90° on the *y *axis in (b')), whereas cell 2 (red) does. Ax, axon; ON, optic nerve; A, anterior, D, dorsal; P, posterior; V, ventral; the asterisk marks cell processes at the apical side of the differentiating RGCs, where the dendrites are forming. The recording was started at 32 hpf; time is shown in hours:minutes. Scale bars, 10 μm. **(c) **Complete sequence of images from cell 2, with images taken every 10 minutes and showing a detail of the basal cell surface dynamics just before axonogenesis. Scale bar, 10 μm. **(d) **Cumulative plot showing the time of axon formation for RGCs with (red dots) and without (green triangles) a visible basal process at the moment of forming the axon, from the 4D analyses *in vivo*. **(e) **Distribution analysis of the delay between apical retraction and axonogenesis in 44 cells followed by 4D imaging.

**Figure 6:**
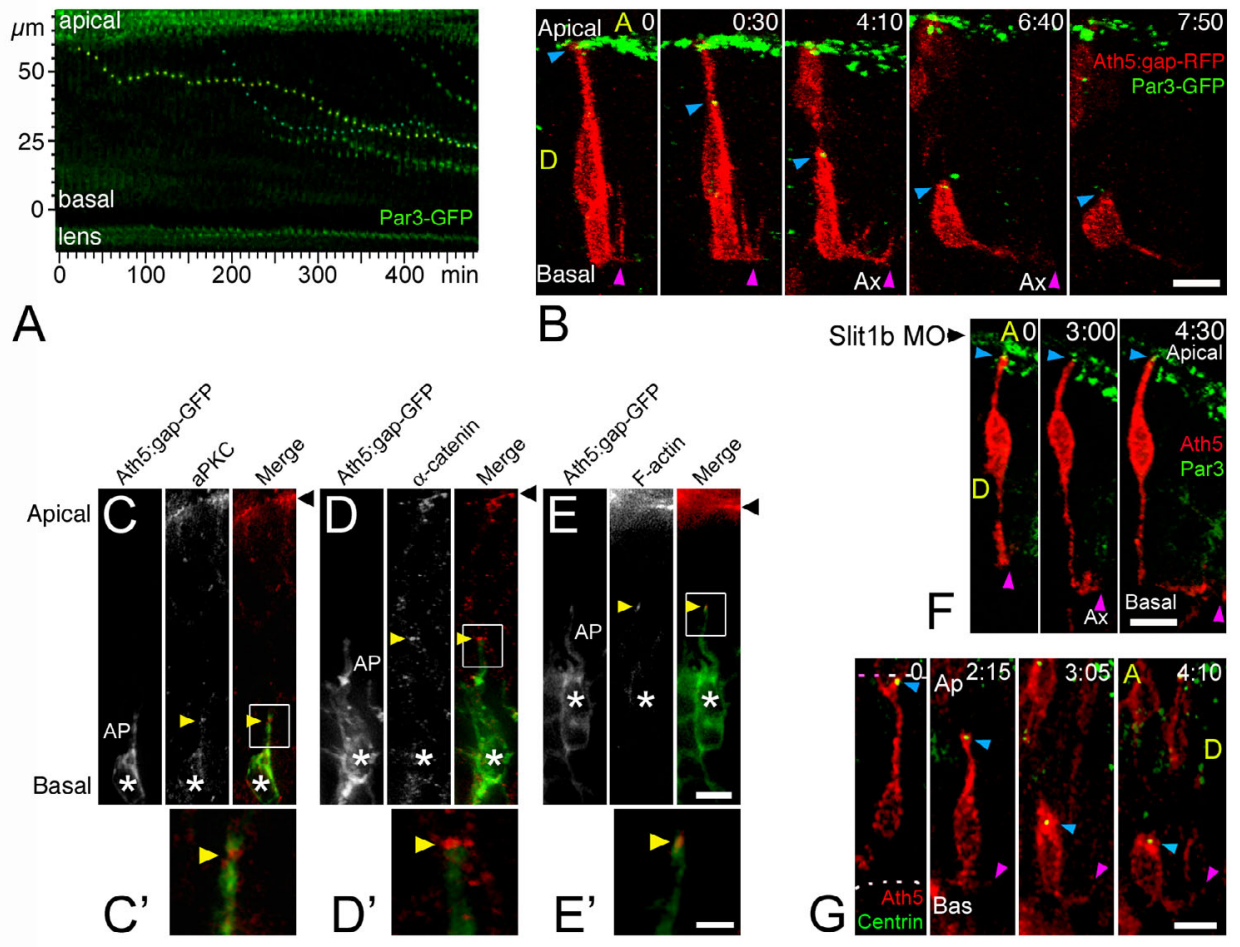
The apical process of retinal ganglion cells (RGCs) remains with apical identity during axon initiation. **(a) **Time-series representation of the Par3-GFP-labeled tips of retracting apical processes. Two dots (from contiguous cells) that show a complete movement from their initial (apical) to their final (basal) positions are pseudo-colored in yellow and blue (Additional file 4). The movement of these dots is reminiscent of that of the retraction of the apical processes (see Figure 3). Time point 0 is at 32 hpf. **(b) **Four-dimensional (4D) analysis of an embryo expressing *ath5:gap-rfp *(injected as plasmid DNA) and Par3-GFP (injected as mRNA). The blue arrowhead indicates the tip of the apical process of a differentiating RGC undergoing axonogenesis, and the purple arrowhead indicates the axon (Additional file 5). The green signal seen at the top of the images represents the Par3-GFP accumulation at the apical border of retinal neuroepithelial cells and of retinal pigment epithelium cells. Time point 0 is at 32 hpf. Ax, axon; A, anterior, D, dorsal; time is shown in hours:minutes. Scale bar, 10 μm. **(c-e) **Immunolabeling of *ath5:gap-gfp *transgenic embryos with different apical markers (anti-aPKC and anti-α-catenin antibodies, and phalloidin-Texas Red to label actin filaments), showing their accumulation (yellow arrowheads) at the tip of retracting apical processes (AP) of RGCs (asterisks). Eight to ten embryos, at different stages, were used in this analysis for each marker, and representative examples are shown. **(c,d) **33 hpf; **(e) **42 hpf. Black arrowhead: apical border of the neuroepithelium. Scale bars, 5 μm (c-e) and 2 μm (c'-e'). **(f) **4D sequence from an *ath5:gap-rfp *(transgenic) and Par3-GFP (mRNA-injected)-expressing embryo, treated with Slit1b morpholino. Note the formation and elongation of the axon (purple arrowhead) without detachment of the apical process from the apical side of the neuroepithelium (blue arrowhead). Time point 0 is at 40 hpf. Ax, axon; time is shown in hours:minutes. Scale bar, 10 μm. **(g) **4D analysis of an embryo expressing *ath5:gap-rfp *(injected as plasmid DNA) and GFP-zcentrin (injected as mRNA). The blue arrowhead indicates the tip of the apical process of a differentiating RGC, and the purple arrowhead indicates the axon (Additional file 6). Some green background appears in the pictures, which mostly comes from cytoplasmic GFP-zcentrin in cells that express a very high level of the fusion protein (this is not the case in the highlighted cell). That the signal is predominantly centrosomal can be seen better in Additional file 6. Time point 0 is at 32 hpf. Ax, axon; A, anterior, D, dorsal; time is shown in hours:minutes. Scale bar, 8 μm.

By using a plasma membrane-targeted form of EGFP under the control of the *ath5 *promoter (*ath5:gap-gfp*), we were able to follow, using 4D-microscopy, the differentiation of RGCs *in vivo *(summarized in Figure 2a). Proliferating precursor cells typically straddle the width of the retinal neuroepithelium. Shortly after their last cell division (I) the cell bodies of RGC precursors move towards the basal side of the neuroepithelium by translocating their nuclei along a basal process that either remains through cytokinesis or extends immediately afterwards (II). Then the apical process, which remains in contact with the apical surface throughout neuroepithelial proliferation, detaches and begins to retract (III to IV; Figure 2b and Additional file 2). In every differentiating RGC analyzed (a total of 65 cells from 13 different embryos), the first neurite forms at the basal surface of the neuroepithelium, in the vicinity of the retinal inner limiting membrane or basal lamina, and immediately differentiates as an axon, forming a growth cone (IV to VI). In these *in vivo *studies, we define the axon as a neurite that grows on the vitreal surface of the retina and is directed towards the optic nerve exit. No other transitory neurites were seen to emerge in any of the observed cases. Just before axon emergence, the cells show highly dynamic filopodia at their basal pole (IV) (Figure 2b,c; see also Figure 6b below) often biased towards the site of the optic nerve exit (Figure 2c). Thus, unlike the situation *in vitro*, there is no evidence of non-oriented (stage 1) or multipolar (stage 2) phases in the polarization of RGCs *in vivo*. Rather, axon emergence is rapid, reproducibly oriented, and happens before the formation of any other neurites.

Previous 4D imaging reveals that dividing neuroepithelial cells in the zebrafish retina often maintain a basal process in contact with the inner surface of the neuroepithelium [21]. Electron microscopic studies by Hinds and Hinds [14] of RGCs differentiating in the mouse retina suggested that the axons of RGCs arise from such basal processes. Without direct time-lapse observations, however, it was not possible for these authors to rule out the possibility that the basal processes of such cells retract before their axons emerge. We therefore took the opportunity offered by 4D imaging to re-examine this question. Until about 40 hpf, growth cones form at the tip of the extended basal process in about half the RGCs examined (Figure 2b,d). In the other half, particularly those adjacent to the inner limiting membrane of the neuroepithelium, the axon emerges without a visible basal process (Figure 2b,b',d; see also Figure 6b below for another example). After 40 hpf, almost all the RGCs form their axons from a basal process (Figure 2d). These results confirm the interpretations of Hinds and Hinds [14] but show that, at early stages, RGC axons need not emerge from an extended basal process.

### Axon outgrowth usually precedes and is independent of apical retraction

**Figure 3:**
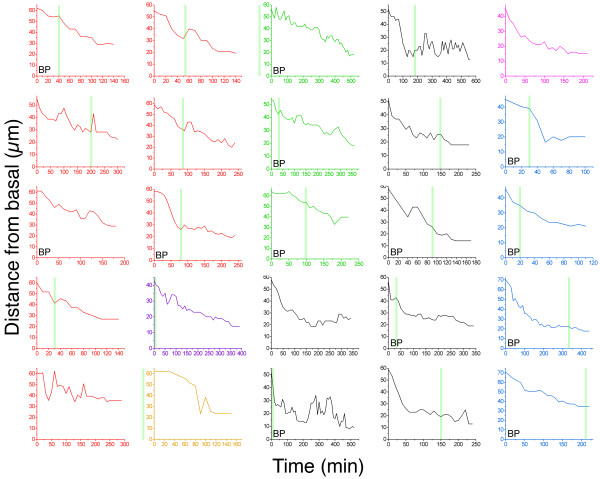
Analysis of the retraction of the apical process. The graphs show the length of the differentiating retinal ganglion cells from the start until the completion of the retraction of the apical process in 25 cells followed by time-lapse *in vivo *(four-dimensional), in seven different embryos. Each color represents cells from the same embryo. The time of axon outgrowth is indicated, when known, as a green line (when the line is outside the graph it indicates only that axonogenesis happened before apical retraction started; it does not show the length of the delay). 'BP' indicates the cells whose axon was formed from a visible basal process. We did not find any obvious correlation between the patterns of apical retraction and the time of axon growth initiation or the presence of a basal process when forming the axon.

**Figure 4:**
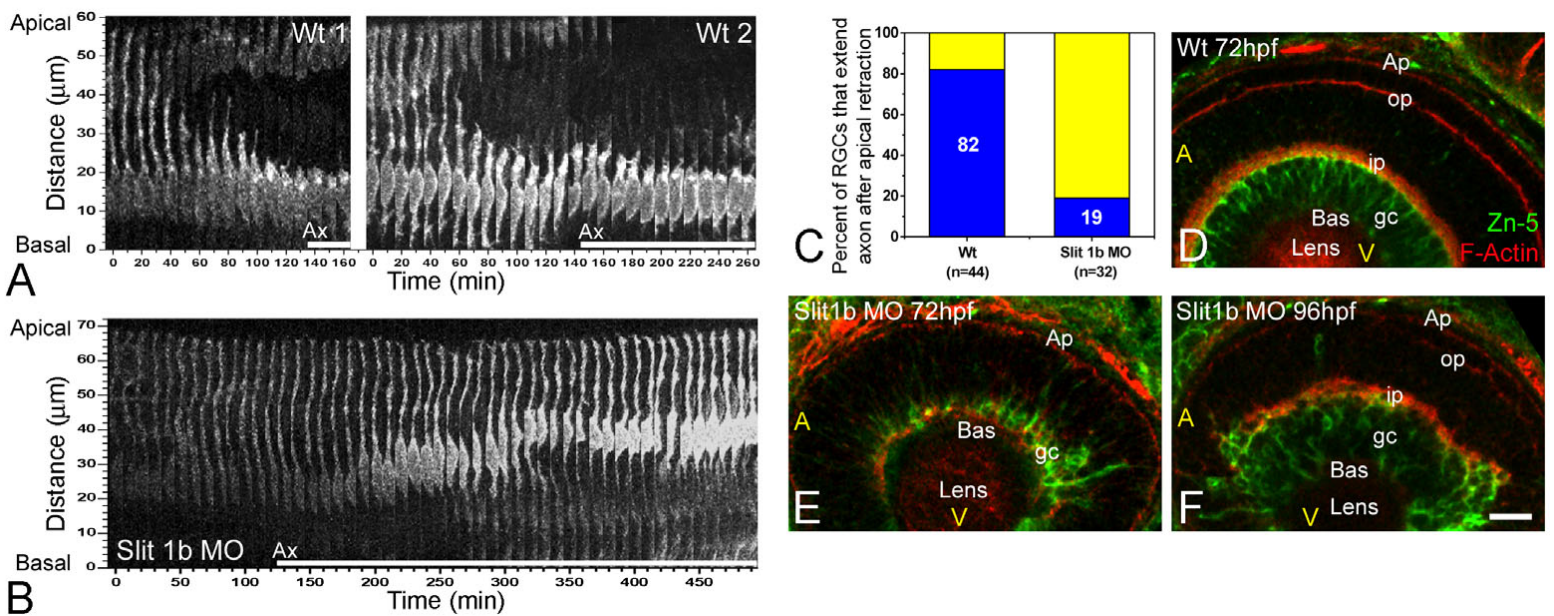
The timing of axon formation does not correlate with the dynamics of apical retraction. **(a) **Time-series images of the cells shown in Figure 2b, showing the retraction of the apical process in a wild-type embryo (also see Figure 3). **(b) **A similar analysis performed in an *ath5:gap-gfp *transgenic embryo, injected with 2 ng of morpholino to Slit1b. The cell is able to extend an axon (not shown in the picture; see Additional file 3) but does not retract its apical process during the time of the movie (more than 8 hours). The age of the embryo at the start of the movie was about 48 hpf. The white bars under the time-series images indicate the presence of an axon (Ax, not seen in the pictures). **(c) **Comparison of the percentage of retinal ganglion cells (RGCs) forming their axons before or after the detachment of the apical process in wild-type and Slit1b morpholino-injected embryos, as analyzed by four-dimensional microscopy; *n *is the number of cells followed in 12 wild-type and 7 morphant embryos. **(d-f) **Parasagittal optical sections of wild-type and Slit1b-treated embryos, labeled for RGCs (gc) with Zn-5, and for F-actin with phalloidin-Texas red, which predominantly stains the inner plexiform (ip) and outer plexiform (op) layers, and the outer limiting membrane (Ap). Bas, basal; A, anterior, V, ventral. Scale bar, 10 μm.

After observing the dynamics of axonogenesis on RGCs *in vivo*, we wondered whether the formation of the axon in RGCs could be related to the retraction of the apical process. To test this, we monitored differentiating RGCs in which the apical process was clearly visible. As shown in Figure 2e, in most of the differentiating RGCs (37 out of 44 cells, from 12 different embryos), the axon does indeed begin to grow after the start of the retraction of the apical process, with a median delay of about 1 hour (29 out of 44 cells presented a delay of between 0 and 2 hours). The time between the onset and completion of the apical retraction (as averaged from 23 cells in which we were able to follow total retraction) was 4.0 ± 2.3 hours (mean ± SD), with a significant variability in the time course of the retraction (Figure 3). Thus, in most RGCs, the axon emerges after the onset of apical retraction but before its completion. In nearly half the cases, the apical processes stop or even re-extend for a short distance (5 to 10 μm) at different moments during the retraction. There are also often minor (1 to 4 μm) back-and-forth oscillations of the apical process before retraction is complete (Figures 3 and 4a). Figure 4a is a set of closely spaced video frames from the apical retraction of the cells shown in Figure 2b and Additional file 2.

**Figure 5:**
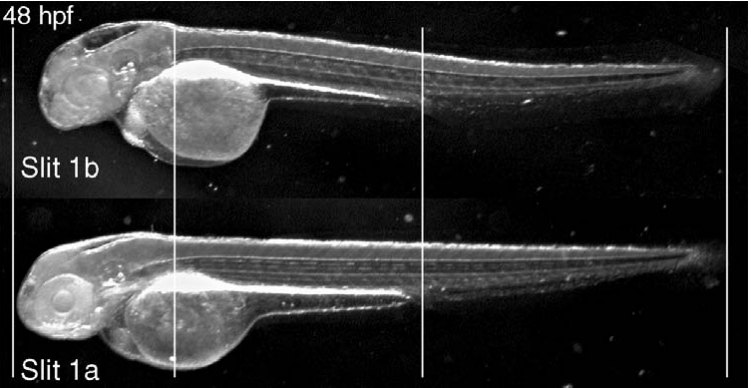
General phenotype of Slit1b morphants. External morphology of a Slit1b morphant at 48 hpf, compared with an embryo treated with the same amount (2 ng) of Slit1a morpholino, which is indistinguishable from a wild-type embryo (not shown). The alignment of the embryos on the vertical lines shows how the general growth of the Slit1b morphants is not affected. The head region, in contrast, seems severely affected, being smaller overall and having smaller eyes and thinner brain walls (with bigger ventricles).

Slit proteins have been proposed as important mediators of axon guidance and neuronal migration [25], and *Slit1b *mRNA is expressed at the developing inner nuclear layer of the retina [26]. Interestingly, mouse mutants for *Slit2 *show occasional abnormal intraretinal trajectories (L Erskine, personal communication). We therefore screened some *Slit *morphants (Figure 5) for defects in RGC polarization. Unexpectedly, we found that embryos treated with Slit1b morpholino show a significant delay in the retraction of the apical process and the migration of the nucleus to the basal side of the retina (Figure 4b). Only a few cells (6 out of 32) in these morphants extend their axons after the initiation of apical process retraction, and in these cases the average delay is shorter than in controls (0.98 ± 0.35 hours versus 1.68 ± 1.4 hours; mean ± SD). In most cells examined (26 out of 32), axonogenesis starts before the beginning of apical process retraction in these morphants (Figure 4c and Additional file 3). This unexpected effect of the *Slit1b *morpholino had the fortuitous benefit of making it obvious that retraction of the apical process is not essential for oriented axon outgrowth.

The effect of the *Slit1b *morpholino on polarization seems temporary. The RGC-specific antibody Zn-5 revealed that in the morphants, RGCs eventually do retract their apical processes and form a normal ganglion cell layer, although they do so much later than their wild-type counterparts (Figure 4d–f).

### During axonogenesis, apical components remain apical

The results described above show that there is no obligatory temporal relationship between the retraction of the apical process and the onset of axon outgrowth and that the axon, even of wild-type RGCs, almost always forms before the apical process completely retracts. This retracting apical process is a remnant of the apical compartment of the neuroepithelial progenitor. *In vitro *studies have shown that molecules normally confined to this apical compartment begin to accumulate in the emerging axon [9]. Thus it might be that *in vivo*, although the morphology shows an apical process at the time of axon emergence, molecularly the situation is similar to what happens *in vitro*. If so, we should be able to test this idea by following apical proteins in RGCs during this transition period.

We decided to investigate this issue by using the fusion protein ASIP/PAR-3-EGFP (Par3-GFP) as an *in vivo *apical marker. This protein was previously shown to accumulate at the apical side of the neural tube epithelium in the zebrafish [20]. Of the three splice isoforms of zebrafish Par-3 described so far [27], this one is the most similar to the 150 kDa Pard-3a, which does not cause retinal patterning defects when overexpressed [28]. Consistent with all these observations, we found that the overexpression of Par3-GFP does not affect retinal lamination or differentiation and that it accumulates at the apical border of the retinal neuroepithelium at early stages. Interestingly, just at the stage at which RGCs start to differentiate, granules containing Par3-GFP move from the apical towards the basal side of the retina (Figure 6a and Additional file 4). They do not, however, travel all the way to the basal surface but seem instead to accumulate throughout the developing neuroepithelium, particularly around the central region, at the stages examined (Additional file 4). Do these Par3-GFP granules, we wondered, remain in the apical processes of differentiating cells? Indeed, 4D analysis of embryos double-labeled with this construct and *ath5:gap-rfp *reveals that these granules dynamically co-localize with the tips of retracting apical processes of RGCs (Figure 6b and Additional file 5). In our experimental conditions, we failed to see an accumulation of this apical marker at the tip of the growing axons (see Figure 6b and Additional file 5). Moreover, antibody staining of differentiating RGCs revealed that other apical markers, such as aPKC, α-catenin and F-actin, remain at the tips of RGCs retracting apical processes (Figure 6c–e). In the Slit1b morphants, apical process retraction is inhibited, and the Par3-GFP signal in RGCs remains at the apical border of the retina even after the formation of the axon (Figure 6f).

Another structure associated with the apical compartment of many epithelia, including the retinal neuroepithelium, is the centrosome [14,29]. To visualize the localization of the centrosome in differentiating RGCs *in vivo*, we generated a fusion protein containing the full zebrafish sequence of the pericentriolar protein centrin [30], attached to EGFP ('GFP-zcentrin'), and used it to follow the subcellular localization of the centrosome in *ath5:gap-rfp*-labeled differentiating RGCs.

We find that GFP-zcentrin labels small dots located at the apical side of the undifferentiated retinal neuroepithelium. Some of these centrosomes are clearly in the apical tips of *ath5:gap-rfp*-positive cells. When these cells enter into the differentiation process that will lead to an RGC, the apical process is retracted as we have described, and the centrosome remains associated with the tip of the retracting process (20 out of 20 cells in five different embryos), even when the cell is extending its axon on the opposite side (Figure 6g and Additional file 6). In all the cases studied, the centrosome approaches the nucleus (on its apical side) only just before the completion of apical retraction. By analogy with what we found for Par3-GFP, we failed to see a basal localization of the centrosome in the differentiating RGCs. Two out of the 20 cells that were followed showed a lateral localization of the GFP-zcentrin-positive centrosome, starting at about 1.5 hours after the apical process had completed the retraction; the rest remained clearly apical.

Taken together, these observations show that, during their differentiation, RGCs undergo a transition phase in which they retain some characteristics of a neuroepithelial cell, such as the apical localization of junctional molecules and the centrosome, while forming the axon. They also show that, at least for RGCs differentiating *in vivo*, the axon emerges basally, whereas apical proteins and the centrosome remain apical.

### The polarity of the neuroepithelium is crucial for RGC orientation

The above experiments show that RGCs polarize in harmony with the neuroepithelium in which they arise. This suggests that the local environment may influence the site of axon outgrowth in differentiating RGCs. If so, axon emergence in RGCs may be affected if the polarity of the retinal neuroepithelium is disrupted, as it is in the zebrafish mutants *nok *and *has*. The *nok *mutant is defective in Pals-1 (also known as Stardust or MPP5), a MAGUK (membrane-associated guanylate kinase) protein that associates with the apical junctional complex. The atypical protein kinase C aPKC-λ, the protein affected in *has *mutants, is a core component of this complex. To investigate how RGC axons emerge in these disrupted environments, we injected the *ath5:gap-gfp *construct or crossed *ath5:gap-gfp *transgenic fish onto these mutant backgrounds.

**Figure 7:**
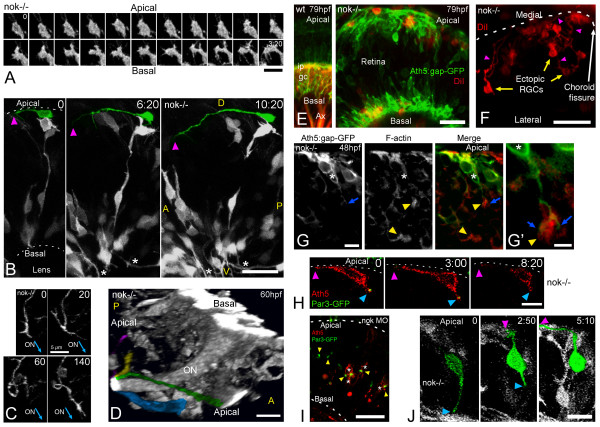
Retinal ganglion cells (RGCs) polarize, but can be inverted in *nok *mutants. **(a) **Complete sequence of images from a three-dimensional (3D) reconstruction, seen from a lateral-apical perspective, of an ectopic RGC in a *nok*^-/- ^retina. Just before axon outgrowth, the RGC presents several filopodia, mostly extending from the site of axon formation (compare with Figure 2c). Scale bar, 5 μm. **(b) **Four-dimensional (4D) analysis of a *nok*^-/-^embryo injected with *ath5:gap-gfp *DNA. The ectopic RGC pseudo-colored in green is extending a long neurite (arrowhead) on the retinal outer surface. Time point 0 is at 32 hpf. Asterisks indicate axons from basally located RGCs; A, anterior, D, dorsal; P, posterior; V, ventral. Scale bar, 25 μm. **(c) **Sequence of 3D reconstructions from a *nok*^-/-^embryo expressing *ath5:gap-gfp*, where an axon growing on the retinal outer surface loops before continuing its growth apparently directed towards the optic nerve (ON) exit. Scale bar, 5 μm. **(d) **3D reconstruction taken from a 4D analysis of a *nok*^-/- ^embryo, transgenic for *ath5:gap-gfp*. The eye is seen from a medio-ventral position. The pseudo-color highlights four ectopic fascicles of axons joining the optic nerve outside the retina (Additional file 7). Scale bar, 10 μm. **(e) **Extended-focus confocal images of *ath5:gap-gfp *transgenic embryos injected with the lipophilic dye DiI into the right tectum to label RGCs retrogradely in the left eye. Scale bar, 15 μm. **(f) **Ventral view of an eye from a *nok*^-/- ^embryo in which RGCs have been retrogradely labeled with DiI. The axons of the ectopic RGCs are seen growing towards the optic nerve (not shown in the picture) on the outer retinal surface. Scale bar, 25 μm. **(g) **Optical section of an *ath5:gap-gfp *transgenic, *nok*^-/- ^retina, stained with phalloidin-Texas red. The 'apical' process (blue arrow) of an ectopic *ath5:gap-gfp *cell (asterisk) ends close to an area where actin filaments appear accumulated (yellow arrowhead). **(g**' **) **Higher magnification of the same cell. Scale bars, 5 μm (g) and 2 μm (g'). **(h) **Sequence of optical sections from a 4D analysis of a *nok*^-/- ^retina expressing *ath5:gap-rfp *and Par3-GFP. The *ath5:gap-rfp*-positive cell is located close to the apical surface of the retina and is extending a neurite on the outer retinal surface (purple arrowhead). On its other end, another process shows an accumulation of Par3-GFP in its tip (blue arrowhead). Time point 0 is at 34 hpf. Scale bar, 10 μm. **(i) **Optical section of a retina from a living *nok*^-/- ^embryo injected with *ath5:gap-rfp *DNA and *par3-gfp *mRNA. The Par3-GFP protein is accumulated in apparently random positions inside the retina, and the *ath5:gap-rfp*-positive cells can be found either on the apical or basal sides of these accumulation points. Scale bar, 20 μm. **(j) **Sequence of extended-focus images from a 4D analysis of a *nok*^-/-^, *ath5:gap-gfp *transgenic embryo, in which an ectopic differentiating RGC (highlighted in green) is seen to retract a basally directed process and then extend an axon on the retinal outer surface (see Additional file 8 for a rotated 3D reconstruction of this cell). Time point 0 is at 32 hpf. Scale bar, 10 μm. All time stamps are in hours:minutes.

A 4D analysis of RGCs in *nok *mutants shows that when *ath5:gap-gfp*-positive cells are located in the normal basal position, they form an axon at the inner surface of the retina and grow towards the site of the optic nerve exit just as in wild-type retinas (Figure 7b). Interestingly, however, many RGCs are malpositioned in *nok *mutants and come to lie at the apical side of the neuroepithelium. These misplaced cells, after showing filopodial activity similar to that seen in normal cells (Figure 7a), also extend a single axon (29 out of 29 cells in 11 embryos). However, the first neurites of these ectopic RGCs are always directed towards the outer retinal surface, indicating a reversal of cell orientation (Figure 7b). Such inversely oriented 'axons' seem to grow more slowly and more erratically than axons from normally positioned RGCs (Figure 7c). Remarkably, however, they also seem to grow towards the site of the optic nerve exit. A time-lapse study of thick confocal stacks (Figure 7d and Additional file 7) reveals that many of the apical axons eventually find and merge with the optic nerve. Because our defined criterion for identifying an axon *in vivo *is difficult to apply in the situation of ectopically differentiating RGCs, we injected the lipophilic dye DiI into the tectum of fixed 79 hpf mutant embryos with the aim of labeling the neurons retrogradely. With this technique, ectopic RGCs and their apical axons are labeled in the same proportion as normal RGCs with their basal axons (Figure 7e,f). Furthermore, *nok*^-/- ^RGCs are not impaired in their abilities to polarize or differentiate when they are grown in dissociated cell cultures (data not shown).

Are these *nok *mutant RGCs polarized in the wrong direction from the beginning of their differentiation process? Previous studies [31] have shown that filamentous actin is normally concentrated at the apical side of the neuroepithelium, but in random central positions in *nok *mutant retinas. Figure 7g shows one of these F-actin accumulations in the proximity of the non-axonal process of an ectopic RGC, suggesting that its polarity is completely inverted; that is, its basally directed process is the equivalent of the apical process in a normal RGC. Consistent with this was our observation, by 4D microscopy, of 12 ectopically differentiating RGCs from 8 different *nok *embryos, which clearly retract this 'apical process' while forming the axon on the retinal outer surface (Figure 7j and Additional file 8). We also looked at the movements of Par3-GFP granules in *ath5:gap-rfp*-positive cells in these mutant retinas (Figure 7i). Figure 7h shows a Par3-GFP granule at the tip of a basally directed 'apical' process in an ectopic RGC, which is extending an axon on the outer retinal surface. However, we never found any accumulation of Par3-GFP at the axon growth cone.

**Figure 8:**
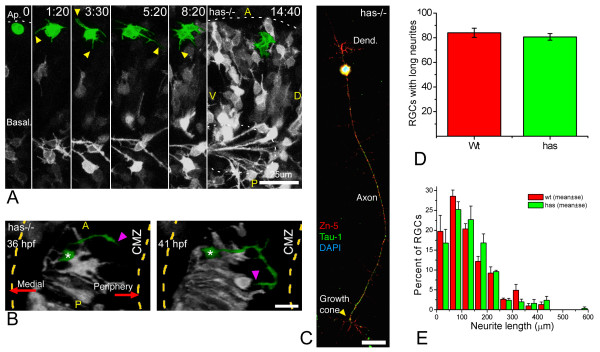
Polarization of ectopic RGCs is impaired in a non cell-autonomous manner in *has *mutants. **(a) **Four-dimensional (4D) analysis of the retina of a *has*^-/- ^embryo injected with *ath5:gap-gfp *plasmid DNA (Additional file 9). The cell highlighted in green divides at the first time frame, and the two daughter cells remain for several hours at the apical region of the retina, increasing their GFP expression but not generating any long neurites. Time point 0 is at 32 hpf. A, anterior, D, dorsal; P, posterior; V, ventral. Scale bar, 25 μm. **(b) **A dorsal view of a three-dimensional reconstruction taken from a 4D confocal analysis of a *has*^-/- ^embryo transgenic for *ath5:gap-gfp*. The ectopic retinal ganglion cell (RGC) highlighted in green is growing a long neurite (arrowheads) that is initially directed towards the retinal periphery (that is, opposite to the optic nerve exit), and then turns back towards the cell. CMZ, ciliary marginal zone. Scale bar, 15 μm. **(c) ***has*^-/- ^RGC in culture, triple-labeled with Zn-5 and anti-Tau-1 antibodies and 4',6-diamidino-2-phenylindole. The cell is indistinguishable from a stage 4 wild-type RGC (see Figure 1). Scale bar, 15 μm. **(d,e) **Quantitative analysis of neuritic outgrowth *in vitro *from *has*^-/-^, compared with wild-type, RGCs (defined as Zn-5-positive cells). **(d) **Number of RGCs with long (more than three cell diameters) neurites; *n *= 200 cells per strain from three independent experiments. **(e) **Distribution of neurite lengths; *n *= 100 neurites per strain (one neurite per cell; the longest was chosen in cells with more than one) from three independent experiments (normalized values).

In *has *mutants, we also found displaced *ath5:gap-gfp*-positive cells. However, in contrast with *nok *mutants, the formation of axons from these putative RGCs was difficult to observe. Most of the apical *ath5:gap-gfp*-positive cells in *has *mutants show a high degree of cell surface activity, and often form several short neurites. In fact, they behave in a way that is reminiscent of RGCs at stage 2 *in vitro *(Figure 8a and Additional file 9). In more extended recording sessions, from *ath5:gap-gfp *transgenics crossed into a *has *mutant background, ectopic RGCs with axons could be seen. Unlike those seen in *nok *mutants, in *has *embryos the ectopic axon-like neurites often do not extend for long distances and many of them grow towards the retinal periphery instead of towards the optic nerve (Figure 8b; see quantification in Figure 10 below). The problems that these cells seem to have in their ability to grow axons or direct them properly could in principle be due to a cell-autonomous effect of the mutation, especially because aPKC activity has been shown to be important for axon determination in rat hippocampal cells [9]. However, this is unlikely to be so, because mutant RGCs positioned on the basal side of the retina form axons normally (Figure 8a and Additional file 9). In addition, *has *mutant RGCs polarize *in vitro *with the same efficiency as wild-type cells (Figure 8c–e). Thus, the differences in polarization between ectopic *has *and *nok *mutant RGCs are more likely to be due to an environmental rather than a cell-autonomous effect.

### Factors extrinsic to RGCs are required for efficient RGC polarization *in vivo*: the role of the RPE and the basal lamina

**Figure 9:**
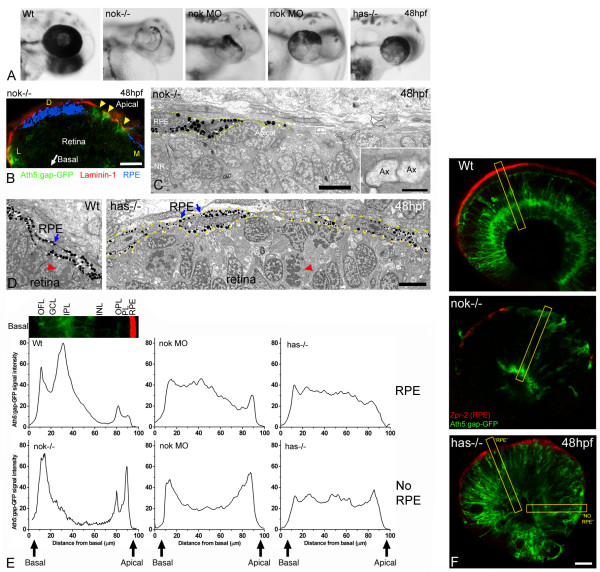
The RPE influences the ability of retinal ganglion cells to polarize in ectopic positions. **(a) **Head region of wild-type, morphant and mutant embryos, showing the distribution of pigment around the retina. Two examples of *nok *morpholino-injected embryos (0.17 pmoles per embryo) are shown. **(b) **Cryosection of the eye from a *nok*^-/-^, *ath5:gap-gfp *transgenic embryo, labeled with an anti-laminin 1 antibody (red). The bright-field image, inverted and pseudo-colored in blue, shows the distribution of the retinal pigment epithelium (RPE). Arrowheads indicate *ath5:gap-gfp*-positive retinal ganglion cells. D, dorsal; L, lateral; M, medial. Scale bar, 20 μm. **(c) **Transmission electron micrograph of the apical region of a *nok *mutant retina, showing the distribution of the RPE cells (dotted lines). Inset, a higher magnification of the boxed region, showing two apparent transverse-sectioned axons (Ax) at the RPE-free apical surface of the retina. Scale bars: low-magnification image, 5 μm; inset, 0.5 μm. **(d) **Transmission electron micrographs of the apical region of a wild-type (Wt) and a *has*^-/- ^embryo. The RPE (blue arrows) is a very organized simple epithelium in the wild type, and it seems disrupted in the *has *mutant. Nevertheless, the picture does not show any actual gap between the RPE cells in the *has*^-/-^embryo. Red arrowheads indicate mitotic cells in a normal (apical) position in the wild-type, and in an ectopic position in the mutant. Scale bar, 5 μm. **(e) **Fluorescence intensity profiles made on confocal sections of 48 hpf *ath5:gap-gfp *transgenic embryos labeled with an anti-RPE antibody (Zpr-2). For the measurements a 20-pixel wide line was drawn along a radius of the retina as in (f). The intensity profile of the green channel (GFP) was plotted and the values were normalized (maximum intensity) and averaged for each embryo; the resulting plots were then normalized to each other (integrated intensity). To compare the profiles in relation to the distribution of the RPE, we positioned the line either in regions where zpr-2 immunoreactivity was detected (RPE) or not (NO RPE). For the *nok *mutants, only measurements of areas without detectable RPE were used. Measurements were made in three to ten different areas from one wild-type, five *nok *mutant, four *nok *morphant and three *has *mutant retinas. The inset picture shows a region of a wild-type retina, like those used for the measurements, which is aligned with the profile plot to show the correspondence with the retinal layers. GCL, ganglion cell layer; INL, inner nuclear layer; IPL, inner plexiform layer; OFL, optic fiber layer; OPL, outer plexiform layer; PL, photoreceptor layer. **(f) **Optical sections of wild-type and mutant retinas of *ath5:gap-gfp *transgenic embryos labeled in red with the Zpr-2 antibody, which stains the RPE. The yellow rectangles show an example of the lines used for the measurements presented in (e). Scale bar, 25 μm.

The results described in the previous section suggest a role of the retinal neuroepithelium environment in the orientation of RGC polarity, but raise the question of which elements of the environment are most important for influencing the site of axon growth in RGCs. A clue to answering this question, we thought, might come from exploring the differences between *nok *and *has *mutants. The most salient difference between these mutants is the pigmentation of the eye (Figure 9a). This suggested to us that the integrity of the RPE could be an important factor. In wild-type embryos, the pigment covers the outer retinal neuroepithelium, whereas in *nok *mutants, large areas of retinal surface are devoid of pigment [32], and in these areas the basal lamina of the RPE, Bruch's membrane, is juxtaposed to the epithelium of the neural retina (Figure 9b,c). In *has *mutants, the gaps in the RPE are rare and small (Figure 9d), with the RPE only seeming really scarce at the peripheral rim.

The RPE-specific antibody Zpr-2 made it possible to compare the distribution of these cells with that of ectopic neurons in the different mutant backgrounds. In *nok *mutants, the ectopic *ath5:gap-gfp*-expressing cells and axons seem to be concentrated in areas of the retina devoid of RPE (Figure 9b,f). In *has *embryos, however, *ath5:gap-gfp*-positive cells seem to be more evenly distributed across the width of the retina (Figure 9f). To quantify this we measured the profiles of *ath5:gap-gfp *fluorescence intensity on confocal sections of these double-labeled retinas. The results, presented in Figure 9e, show that the accumulation of label at the inner side of the retina in the wild-type retina is converted into an accumulation of signal at both retinal surfaces in the *nok *mutant retina. In *nok *morphants (which show a milder *nok*-like phenotype) there is clearly a small peak of apical *ath5:gap-gfp *signal in areas where the RPE is absent. These data are consistent with the RGCs being close to the surface on which they are growing axons. This may be so, because once the axon forms, it is likely to exert enough tension on the cell body to tow it towards the relevant surface. In *has *mutants, although the RPE covers most of the retinal apical surface, there are only few areas devoid of RPE. In these areas we also found a slightly higher apical *ath5:gap-gfp *signal.

**Figure 10:**
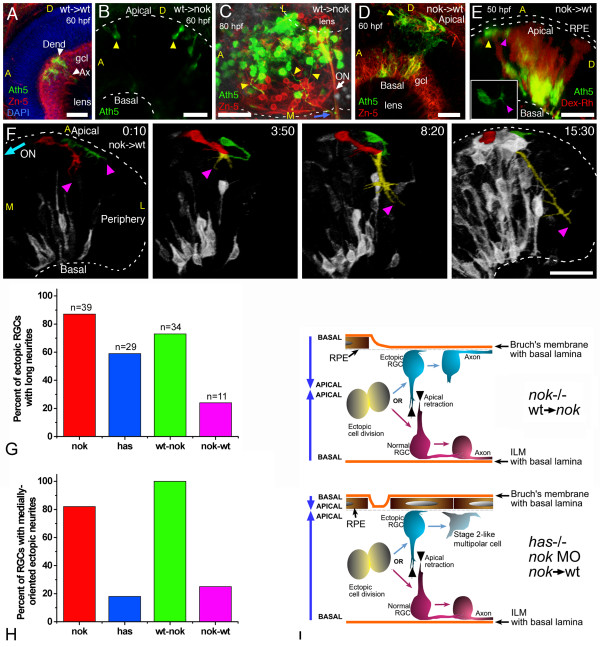
Importance of the tissue environment for the differentiation and orientation of ectopic retinal ganglion cells (RGCs). **(a) **Extended-focus confocal image from a wild-type host embryo transplanted with blastomeres from an *ath5:gap-gfp *transgenic wild-type embryo. gcl, ganglion cell layer. **(b,c) **Extended-focus images from *nok*^-/- ^host retinas transplanted with *ath5:gap-gfp *transgenic cells (wild type). In (b), the lateral view of the eye shows the presence of ectopic *ath5:gap-gfp*-positive cells (arrowheads); in (c), the eye is seen from a ventral view, and many ectopic (apical) donor RGCs that extend axons (yellow arrowheads) joining the optic nerve (ON) on the outside of the eye are shown. **(d-f) **Images of wild-type hosts transplanted with blastomeres from *nok*^-/-^, *ath5:gap-gfp *transgenic donors. **(d) **Extended-focus confocal image, in which the donor RGCs (positive for both Zn-5 (red) and *ath5:gap-gfp *(green)) are found either mixed with host RGCs in a normal-appearing ganglion cell layer (gcl) or on the apical side of the retina. **(e) **Rotated extended-focus confocal image, showing how a *nok*^-/- ^donor-derived ectopic (apical; yellow arrowhead) *ath5:gap-gfp*-positive cell starts to extend a neurite (purple arrowhead) that will grow only a few micrometers before turning back to the originating cell (Additional file 10). The red stain shows all the donor cells, labeled with dextran-tetramethylrhodamine β-isothiocyanate (Dex-Rh). No transplanted cells are detected in the surrounding retinal pigment epithelium, indicating that it must all be of wild-type (host) origin. The inset shows a better view (from the apical side, in this case in an optical section orthogonal to the confocal laser) of the cell in the same time point. **(f) **Sequence of rotated three-dimensional reconstructions taken from a four-dimensional analysis. The two cells highlighted are from the donor (*nok*^-/-^) and are extending long neurites, which will grow together towards the retinal periphery (colored yellow where they are indistinguishable from each other). Time point 0 is at 32 hpf. ON, optic nerve; A, anterior; D, dorsal; L, lateral; M, medial. All scale bars are 25 μm long. **(g) **Comparison of the number of apically located *ath5:gap-gfp *cells extending a long neurite (longer than three cell diameters), in different mutant and morphant conditions. Total number of cells (*n*) and embryos analyzed (cells/embryos): *nok*^-/-^, 39/11; *has*^-/-^, 29/8; transgenic *nok*-wild-type, 34/9; transplanted wild-type-*nok*, 11/6. **(h) **The proportion of these cells in which the neurite is growing towards the medial region of the eye, where they would be able to meet the optic nerve. **(i) **Summary of the observed phenotypes of ectopic RGCs in mutant, morphant and transplantation conditions.

These results suggest that access to a basement membrane, either the inner basal lamina or the neural retina or the outer Bruch's membrane of the RPE, may be sufficient to elicit oriented axon emergence. If this is so, then ectopic apical RGCs that are prevented from gaining access to Bruch's membrane should have trouble polarizing, whereas even wild-type RGCs should show reverse polarization if given access to this basement membrane. To test this hypothesis we performed blastomere transplantation experiments between wild-type and *nok *mutant embryos. As expected from previous results, some transplanted cells from *ath5:gap-gfp*-positive wild-type donors into *nok *hosts seem ectopically localized (Figure 10b). These wild-type cells are generally reverse-polarized and grow axons on the outer retinal surface, directed towards the optic nerve exit (Figure 10c). When *nok *mutant cells are transplanted into wild-type embryos, small clones of mutant *ath5:gap-gfp*-positive cells behave like normal RGCs (not shown), as described previously [31]. However, when the transplanted clones are larger, there is a clear local disruption of the host's neuroepithelial polarity (Figure 10d). In most of these cases the neural retinal clones are next to wild-type intact RPE (Figure 10e and Additional file 10), resulting in ectopic *nok *mutant RGCs with no access to Bruch's membrane. What is particularly telling here is that most of these ectopic RGCs either fail to grow long neurites or grow axons that are misdirected (Figure 10f).

The graphs in Figure 10g–h show a quantitative comparison of directed outgrowth of axons from ectopic RGCs in *nok *and *has *mutants, compared with that of ectopic transplanted RGCs in mosaics. The data support the hypothesis that the inversion of ectopic RGC orientation in *nok *mutants is dependent on the absence of the RPE. To test this further, we used low doses of a translation-blocking *nok *morpholino (0.17 to 0.20 pmoles per embryo; Figure 9a), which, although able to produce ectopic RGCs, are not efficient at removing the RPE. Thus, ectopic RGCs in these embryos are usually apposed to the RPE rather than Bruch's membrane. According to the hypothesis being tested, these cells should also have difficulty in polarizing. Indeed, these ectopic RGCs do not generally orient in either the normal or reverse direction, and the axons of these cells, when present, tend to grow aberrantly, often towards the retinal periphery (Additional file 11). A summary of the observed behaviors of RGCs in the analyzed conditions is presented in Figure 10i.

## Discussion

Intrinsic mechanisms are used predominantly to break symmetry in neurons that develop in a two-dimensional symmetric *in vitro *environment, but *in vivo *the polarized three-dimensional environment provides extrinsic signals that orient differentiating neurons. To approach the mechanisms that drive neuronal polarization *in vivo*, we have used 4D microscopy to examine how RGCs in zebrafish make the morphological transition from post-mitotic neuroepithelial-shaped cells to neurons with basally oriented axons. Comparison of the polarization of mammalian hippocampal neurons, or indeed zebrafish RGCs *in vitro*, shows that there are crucial differences between RGC polarization in culture and in the living tissue. These differences may be explained on the basis of environmental influences. Cultured cells are in an almost completely artificial environment. They are in contact with a flat substrate (often rich in laminin) and rarely come into contact with other cells. *In vivo*, however, differentiating neurons are naturally almost completely surrounded by cells with which they interact extensively. In addition, the native environment provides heterogeneously distributed positional cues that are difficult to emulate *in vitro*.

These differences are likely to influence the initial stages of differentiation. *In vitro*, the cell is rounded and extends pseudopodia and filopodia all around its free surface, whereas *in vivo *the newborn RGC first extends from the apical to the basal surface of the neuroepithelium assuming a spindle shape, typical of neuroepithelial cells, and then starts retracting its apical process. RGCs *in vivo *show filopodial activity at the basal side for 1 or 2 hours before axonogenesis, whereas RGCs differentiating in culture extend short neurites at several points of their cell bodies, before one of them starts to elongate in an axon-like manner. This seems very comparable to stage 2 of rat hippocampal neurons differentiating *in vitro*, although we found that RGCs, in our culture conditions, do not usually present a multipolar morphology, but just an alternative growth and retraction of neurites at different points. Another difference from rat hippocampal cells is that zebrafish RGCs seem to form their dendrites *de novo *in the transition between stages 3 and 4. The second stage, which can last up to several hours in cultured RGCs, is not seen in normal differentiating RGCs *in vivo*, because these cells seem to pass directly from a neuroepithelial-like spindle-shaped cell to one in which the RGC has only a single fast-extending neurite, which invariably becomes the axon. This neurite is always formed at the basal pole of the cell, opposite to a retracting apical process. Signals distributed heterogeneously in the retinal neuroepithelium could be responsible for restricting axon formation to the basal side [33]. A similarly restricted cellular behavior has very recently been described for HSN motorneurons differentiating in living *C. elegans *larvae [34].

The seminal work of Hinds and Hinds [14] in the developing mouse retina, following the much earlier observations of Ramón y Cajal [13], suggested that in RGCs the axon and the basal process were the same thing, or that the axon was formed from the basal process. Recent time-lapse observations of bipolar cells *in vivo *showed that the neurites extending into the outer plexiform layer and the inner plexiform layer develop respectively from the unretracted apical and basal processes of the migrating precursor [35]. In the present study, 4D imaging allowed us to observe the dynamics of axonogenesis in RGCs *in vivo *and to see that in many cases, especially at early stages, the axon emerges directly from the cell body after it reaches the basal surface of the neuroepithelium and is in apposition to it. It is interesting that the filopodial activity at the basal side of such differentiating RGCs starts before the extension of the axon, similar to what was previously described for differentiating rat RGCs [36]. At later stages, however, most RGC axons do seem to grow from a basal process. The reason why basal process-free differentiating RGCs are only seen at relatively early stages of retinal differentiation may reflect the fact that the first RGCs are able to translocate their somas all the way to the basalmost position, whereas later-differentiating somas are blocked from reaching the basal surface by a layer of tightly packed RGCs and must form axons from a distance (see Figure 2a).

We wondered whether the retraction of the apical process or components that are normally localized to this process provide intrinsic information that tells the differentiating RGC when and where to form the axon. In the retina, post-mitotic RGC precursors transiently acquire a spindle-like, neuroepithelial morphology. They begin to lose this neuroepithelial morphology with the retraction of the apical process, and the axon is usually formed after this retraction has started but has not yet completed. However, our observations also show that axons can emerge from RGCs before retraction of the apical process begins. In Slit1b morphants, most RGCs form axons before apical process retraction begins. Our results with Slit1b are consistent with previous work showing a role for Slit proteins in neuronal migration [25], and it will be very interesting to investigate how Slit1b is involved in apical retraction, although that would have to be the subject of another study. All these observations indicate that axon extension occurs independently of the timing of apical retraction.

It has been confirmed by different groups that the Par-3-Par-6-aPKC complex accumulates at the tip of the growing axon of cultured hippocampal neurons, where it has a fundamental role in the determination of axon identity [7,9]. We did not find detectable accumulation of Par3-GFP in the RGC axons forming *in vivo*. Rather, this protein, although present in differentiating RGCs, remains apical. This finding supports the apparent absence of a role for apical complex components in mushroom body neurons during axonogenesis (or dendritogenesis) in living *Drosophila *larvae [12]. We also show that aPKC-λ-deficient (*has*^-/-^) RGCs are able to polarize efficiently and extend axons *in vivo *when these cells are in contact with the inner surface of the retina and that they can also polarize *in vitro*. In this case it may be that because the *has *mutation affects aPKC-λ and not aPKC-ζ, the latter compensates for the absence of the former and that double mutants may be needed to reveal whether aPKCs are necessary for oriented RGC axon outgrowth *in vivo *and intrinsic polarization *in vitro*.

It was reported recently that the localization of the centrosome is involved in establishing the site of axon emergence from cultured rat hippocampal cells [11]. The authors of that study suggested that during telophase of a neurogenic apicobasal cell division [37-39], the centrosome of the basal post-mitotic daughter cell, being located opposite to the apical surface, would determine the emergence of the axon from the basal side. However, this could not work in our case because there are no apicobasal cell divisions in the differentiating zebrafish retina, and RGCs originate from planar oriented divisions [17,21]. The absence of apicobasal divisions in the zebrafish retina is also consistent with our observation that RGC precursors inherit components such as Par3-GFP, aPKC, α-catenin and F-actin of the apical adhesion complexes in the tips of their retracting apical processes. Surprisingly, through the dynamic analysis of RGC differentiation by 4D microscopy, we also found that the centrosome does not move from its apical position during the whole process of axonogenesis. This observation does not exclude the possibility of an essential role of this organelle in axon determination or axon growth but shows that its localization close to the site of axon formation is not necessary for the normal polarization of zebrafish RGCs *in vivo*. In addition, our data support previous data obtained from the ultrastructural analysis of the differentiating mouse retina [14] and other systems, including hippocampal pyramidal cells [40,41]. Many studies have shown that the centrosome, which is essential for nuclear translocation, is located on the leading edge side of the nucleus in migrating neurons [42]. In our system, the centrosome localizes to the trailing edge as the nucleus translocates towards the basal surface. This difference could be because RGCs do not actually migrate but just elongate and translocate their nuclei.

We have used two mutations, *nok *and *has*, to analyze the role of neuroepithelial polarity in the orientation of RGC polarity *in vivo*. We found in *nok *mutants that ectopic RGCs are able to polarize properly (that is, to form only one axon) but that their orientation is often inverted (that is, the axon is directed towards the outer retina). In accordance with previous findings that apical markers, including F-actin and Par-3, are accumulated in random positions inside the retina in these mutants [28,31], we found that basally directed processes of these ectopic differentiating RGCs express Par3-GFP in the proximity of F-actin staining. In the *nok *mutant retinas there are large gaps where the RPE is not present and where Bruch's membrane, the basal lamina of the RPE, becomes apposed to the surface of the retinal neuroepithelium. In these regions the neuroepithelial polarity seems completely inverted (that is, the apical complex is basally located with respect to a basal lamina present at the apical surface of the neuroepithelium).

We also unexpectedly found that there were fewer ectopic RGCs concentrated on the apical side of the retina, as well as many fewer ectopic axons in *has *mutants than in *nok *mutants. The RPE is more intact in *has *mutants. This observation suggests that the RPE may normally inhibit reverse polarization, either by generating an inhibitory signal or by blocking access to permissive signals on Bruch's membrane. It has previously been shown in chick and quail that a small proportion of axons naturally escape from the optic nerve layer and grow between the cells of the RPE and Bruch's membrane, and that the Bruch's basal lamina is a good substrate *in vitro *for RGC axon growth [43]. This favors the plausibility of the second hypothesis. In addition, inversion of RGCs has been observed in organ-cultured retinas in which the vitreal surface was exposed to chondroitin sulfate [44]. In this case, too, ectopic RGCs extend axons on the outer surface of the retina. Many previous studies have proposed that the RPE has an essential role in retinal lamination [31,45-47], and it is tempting to speculate that our observations could also help explain their results.

## Conclusion

The polarity of the neuroepithelium seems to be a major determinant in the site of axon outgrowth from RGCs. Our work suggests that there is an intrinsic tendency for RGCs to polarize (to form only one axon) but that RGCs differentiating *in vivo *use signals, like those normally found at the inner limiting membrane of the retina, to define the orientation of this polarization and the position of axon emergence.

## Abbreviations

4D = four-dimensional; aPKC = atypical protein kinase C; CCD = charge-coupled device; EGFP = enhanced green fluorescent protein; *has *= *heart and soul*; hpf = hours after fertilization; mAb = monoclonal antibody; mRFP = monomeric red fluorescent protein; *nok *= *nagie oko*; pAb = polyclonal antibody; PBS = phosphate-buffered saline; PCR = polymerase chain reaction; RGC = retinal ganglion cell; RPE = retinal pigment epithelium.

## Competing interests

The author(s) declare that they have no competing interests.

## Authors' contributions

FRZ designed, performed and analyzed most of the studies described. LP performed the design and cloning of the Ath5:gap-GFP construct and the generation of the correspondent transgenic fish, helped in designing the Ath5:gap-RFP construct and performed the *in vivo *4D study of GFP-zcentrin/Ath5:gap-RFP expression. CJW designed and cloned the GFP-zcentrin construct and helped in designing the Ath5:gap-RFP construct. C-BC designed and provided the morpholinos to Slit proteins. WAH conceived of the study and participated in the design of most of the experiments. All authors read and approved the final manuscript.

## Supplementary Material

Additional file 1 A Quick Time video file showing *ath5:gfp*-positive RGCs differentiating *in vitro*. a and b are two GFP-expressing cells that undergo the initial stages of differentiation in culture. Fluorescence is shown only at the beginning and the end of the movie. Time is shown in hours:minutes:seconds. Click here for file

Additional file 2 A Quick Time video file showing RGC differentiation *in vivo *in an embryo injected with *ath5:gap-gfp *plasmid DNA, to obtain a mosaic expression. Two differentiating RGCs are marked as 1 and 2. The arrowheads point to the tips of the apical process and of the elongating axon. The asterisk marks the differentiation of the dendrites in cell 2. Stage at start is 32 hpf. Time is shown in hours:minutes:seconds. Click here for file

Additional file 3 A Quick Time video file showing the effect of Slit1b morpholino injection on RGC differentiation. The embryo is transgenic for Ath5:Gap-GFP. The arrow points to the cell body of an RGC that forms an axon marked with the arrowhead. Stage at start is 48 hpf. Time is shown in hours:minutes:seconds. Click here for file

Additional file 4 A Quick Time video file showing localization of Par3-GFP fusion protein in a zebrafish embryo retina during RGC differentiation. The arrowheads mark two different Par3-GFP granules seen to move from the apical border of the neuroepithelium. Stage at start is 32 hpf. Time is shown in hours:minutes:seconds. Click here for file

Additional file 5 A Quick Time video file showing RGC differentiation in an embryo double-labeled with Par3-GFP (mRNA, ubiquitous expression) and Ath5:Gap-RFP (DNA, mosaic expression). Blue arrowhead: tip of the retracting apical process of a differentiating RGC. Pink arrowhead: tip of the elongating axon from the same cell. Stage at start is 32 hpf. Time is shown in hours:minutes:seconds. Click here for file

Additional file 6 A Quick Time video file showing RGC differentiation in an embryo double-labeled with GFP-zcentrin (mRNA, ubiquitous expression) and Ath5:Gap-RFP (DNA, mosaic expression). Several RGCs are seen to start retracting their apical processes, most of them clearly showing a centrin-GFP-labeled centrosome at their tips. Stage at start is 32 hpf. Time is in minutes. Click here for file

Additional file 7 A Quick Time video file showing ectopic axon growth in a *nok *mutant/*ath5:gap-gfp *transgenic embryo. Ventral view of the retina and optic nerve, taken from a 4D movie, composed of thick stacks, that was rotated through 90°. Stage at start is 48 hpf. Time is shown in hours:minutes:seconds. Click here for file

Additional file 8 A Quick Time video file showing retraction of an ectopic RGC's basally directed process in a *nok *mutant/*ath5:gap-gfp *transgenic embryo. The RGC marked with an arrow is differentiating close to the apical surface of a *nok *mutant retina. The arrowheads show a retracting process, directed basally, and the tip of a neurite extending on the apical surface, from the same cell. Stage at start is 48 hpf. Time is shown in hours:minutes:seconds. Click here for file

Additional file 9 A Quick Time video file showing failure to differentiate of apical cells expressing Ath5:Gap-GFP in a *has *mutant retina. The cell pointed with an arrowhead at the start of the movie will divide ectopically to give rise to two daughter cells (a1 and a2). Cell a2 will eventually form an axon at the retinal basal surface, but cell a1 will move towards the apical surface, where it will divide again. Both a1's daughter cells (a1' and a1") will remain in this apical position, increasing GFP expression (an indicator of RGC fate), but none of them will form an axon during the recording time. A similar example is shown as cell b (dividing as b1 and b2). Stage at start is 32 hpf. Time is shown in hours:minutes:seconds. Click here for file

Additional file 10 A Quick Time video file showing failure to grow long neurites of a *nok *apical cell in a wild-type environment. The cell marked with an arrowhead derives from a *nok *mutant/*ath5:gap-gfp *transgenic embryo whose blastomeres were transplanted into a wild-type host (unlabeled). At time point 16 minutes 49 seconds, a double-labeled rotating three-dimensional reconstruction is shown, in which, in addition to the GFP, the red signal from dextran-rhodamine present in all transplanted cells is shown. This reconstruction shows how the marked cell is located on the retinal apical surface, and that it is not in contact with mutant transplanted RPE cells (which would be labeled red). The cell grows only a short neurite that turns back to the cell body and does not extend further during the recording. Stage at start is 32 hpf. Time is shown in hours:minutes:seconds. Click here for file

Additional file 11 A Quick Time video file showing that in *nok *morphants, RGCs can differentiate ectopically but fail to direct the axons towards the optic nerve exit. An apical RGC (arrow) is shown to have extended a very long neurite (arrowhead) on the apical retinal surface but, instead of being directed towards the back of the eye (where it would meet the optic nerve), it is directed in an opposite direction, towards the retinal periphery. Stage at start is 48 hpf. Time is shown in hours:minutes:seconds. Click here for file
